# The clinical potential of microRNAs

**DOI:** 10.1186/1756-8722-3-37

**Published:** 2010-10-06

**Authors:** Anuradha Budhu, Junfang Ji, Xin W Wang

**Affiliations:** 1 Liver Carcinogenesis Section, Laboratory of Human Carcinogenesis, Center for Cancer Research, National Cancer Institute, Bethesda, MD, USA

## Abstract

MicroRNAs are small noncoding RNAs that function to control gene expression. These small RNAs have been shown to contribute to the control of cell growth, differentiation and apoptosis, important features related to cancer development and progression. In fact, recent studies have shown the utility of microRNAs as cancer-related biomarkers. This is due to the finding that microRNAs display altered expression profiles in cancers versus normal tissue. In addition, microRNAs have been associated with cancer progression. In this review, the mechanisms to alter microRNA expression and their relation to cancer will be addressed. Moreover, the potential application of microRNAs in clinical settings will also be highlighted. Finally, the challenges regarding the translation of research involving microRNAs to the clinical realm will be discussed.

## Review

### The Biogenesis and Physiological Functions of MicroRNAs

MicroRNAs are a group of small noncoding functional RNAs that are approximately 22 nucleotides in length [1]. MicroRNAs are transcribed by RNA polymerase II or III as longer primary microRNAs termed pri-microRNA. This molecule is then modified in the nucleus through capping and polyadenylation and subsequently cleaved into smaller segments by Drosha, an RNAseIII enzyme. This forms a hairpin precursor of approximately 60-70 nucleotides, termed pre-microRNA, which is exported to the cytoplasm and modified by another enzyme, the RNAseII endonuclease, Dicer, to form a duplex of mature microRNA. One of the microRNA strands of the duplex is loaded onto the RNA-induced silencing complex (RISC) where it is then able to either cleave RNA targets or repress protein translation dependent upon its complementarity to the target mRNA. Through their binding to target mRNA sequences, microRNAs have a large number of biologically diverse functions. They have the capacity to control the expression of many downstream genes which can affect several cell regulatory pathways, such as cell growth, differentiation, mobility and apoptosis.

### The Detection of MicroRNA Expression

Several techniques have been developed to examine microRNA expression. One of the most predominant methods in the literature is microRNA microarrays. Microarray technology offers a powerful high-throughput tool to monitor the expression of thousands of microRNAs at once [2]. Quantitative reverse transcription-polymerase chain reaction (qRT-PCR) is another reliable and highly sensitive technique for microRNA detection, which is simple and robust, and only requires very small amounts of input total RNA [3]. Standard northern blotting has also been employed to detect and validate microRNA expression levels [4]. In addition, techniques are available to detect microRNAs by *in situ *hybridization. Although various microRNAs have been detected from tissue sources, these methods require invasive techniques to collect the starting material. Therefore, procedures have also been established to measure microRNA expression in blood products to enable clinical feasibility of microRNA measurement [5]. Most recently, the advent of next generation sequencing technologies allows for the measurement of the absolute abundance as well as the discovery of novel microRNAs. These various techniques have allowed researchers to measure the levels of many microRNAs and determine how alterations in their expression level are associated with particular phenotypes and how they can be clinically utilized. These aspects of microRNA expression levels are discussed in the remainder of this review.

### The Role of MicroRNAs in Cancer

Since their discovery in nematodes, microRNAs have been shown to play a role in cancer (Table 1). The expression patterns, function and regulation of microRNAs in normal and neoplastic human cells are largely unknown but emerging data and their frequent location at fragile sites, common break-points or regions of amplification or loss of heterozygosity reveal that they may play significant roles in human carcinogenesis. Other possible mechanisms of altered microRNA expression include defective microRNA processing or post-transcriptional regulation, germ-line or somatic mutation and epigenetic changes such as methylation [6-9]. The abnormal expression of several microRNAs have been observed in Burkitt's lymphomas, B cell chronic lymphocytic leukemia (CLL) and in many solid cancer types, including breast, liver, lung, ovarian, cervical, colorectal and prostate [10-21]. Functional analysis has revealed the downregulation of PTEN by miR-21, the tumor suppressor function of the let-7 family and the oncogenic function of the miR17-92 cluster [22-24]. The biological and clinical relevance of microRNA expression patterns have been established in human B cell CLL and solid tumors, including breast cancers [11,15,25].

**Table 1:** MicroRNAs Associated with Cancer

Cancer Type	Human microRNA	Potential Function	References
Breast Cancer	miR-21, miR-125b; *miR126*, *miR-206*, *miR-335*	OncomiR; *Metastasis Suppressor*	[49,75,76]

Colon Cancer	miR-21; *miR-34a*	OncomiR; Tumor Suppressor	[41,77-79]

Lung Cancer	miR-21, miR17-92 cluster, miR-106b/93/25 cluster; *Let-7a*, *miR-143*, *miR-145*	OncomiR; Tumor Suppressor	[13,40,80,81]

Pancreatic Cancer	miR-196a, miR196b	OncomiR	[82-84]

Prostate Cancer	miR-21, miR-15/16; *miR-145*, miR-146, *miR-330*, *miR-205*	OncomiR; *Tumor Suppressor*	[69,85,86]

Ovarian Cancer	miR-141, miR-200a/b/c; *miR-199a/b*, *miR-140*, *miR-145*, *miR-204*, *miR-125a/b*,	OncomiR; *Tumor Suppressor*	[87,88]

Hepatocellular Carcinoma	miR-21, miR-224, miR-34a, miR221, miR-222, miR-106, miR-303; *miR26a/b*, *let-7g*, *miR-122*, *miR-422b*, *miR-145*, *miR-199*	OncomiR; *Tumor Suppressor; Metastasis*	[46,52,53,89,90]

Thyroid Cancer	miR-146, miR-221, miR-222, miR-181b, miR-155, miR-224	OncomiR	[91-93]

Each microRNA has the distinct capability to potentially regulate the expression of hundreds of coding genes and thereby modulate several cellular pathways including proliferation, apoptosis and stress response [26]. Their altered expression in cancer can be a causative factor or perhaps a consequence of the disease state. Dependent upon the nature of their target gene(s), microRNAs may function as tumor suppressors by downregulating target oncogenes (e.g. let-7 g, miR-15/16 and miR-34) or as oncogenes by negatively controlling genes that regulate tumor cell differentiation and apoptosis (e.g. miR-155 and miR-21) [27]. Alternatively, changes in microRNA expression may be a downstream effect of potent oncogenes or tumor suppressors in the carcinogenesis process such as the modulation of miR-34 by p53 [28]. MicroRNAs have also been shown to play a role in cancer progression through the modulation of cellular adhesion, cell matrix and signaling activities [29-33]. In addition, microRNAs play roles in regulating the expression of hypoxia-related genes, vascular endothelial growth factors [34-36].

### The Clinical Applications of MicroRNAs

Since the expression of microRNAs are altered in cancers, it is thought that they may function as suitable biomarkers for disease state and progression. Recent studies indicate that expression profiling of microRNAs is a superior method for cancer subtype classification and prognostication [10,11,20]. The application of microRNA screening for the purposes of diagnosis and prognosis are briefly described below.

#### Diagnostic MicroRNAs

Multiple reports have noted the utility of microRNAs for the diagnosis of cancer [37,38]. microRNA expression profiles have been used to distinguish tumor from normal samples, identification of tissue of origin for tumors of unknown origin or in poorly differentiated tumors and to distinguish different subtypes of tumors. Sample datasets have been stratified to show that certain alterations of microRNAs occur in patients at an early stage of cancer and thus may be quite useful for early detection. Large tissue specimens are not needed for accurate MicroRNA detection since their expression can be easily measured in biopsy specimens. Although the majority of these studies have used tissue to assess microRNA levels, recent studies have shown that microRNAs can be measured in formalin fixed paraffin embedded (FFPE) tissues [39]. Given the invasive nature of fresh/frozen tissue collection and the availability of FFPE, this serves as a major advance in the feasibility of measuring microRNA levels for the purposes of diagnosis. Recent studies have also shown that microRNAs can be detected in serum. These studies offer the promise of utilizing microRNA screening via less invasive blood-based mechanisms. In addition, mature microRNAs are relatively stable. These phenomena make microRNAs superior molecular markers and targets for interrogation and as such, microRNA expression profiling can be utilized as a tool for cancer diagnosis [10,40].

#### Prognostic MicroRNAs

The potential clinical utility of microRNA extends beyond the realm of diagnosis to other important clinical measures such as prognosis and treatment response. A series of publications has shown that microRNAs are useful indicators of clinical outcome in a number of cancer types [10,40-45]. In addition, microRNAs have been shown to play a predictive role in determining the tendency for recurrence and metastasis [46-50]. These microRNA alterations have not only been found in tumor specimens, but have also been observed in surrounding non-cancerous tissue, indicating that microRNAs may also serve to detect alterations in the cancer microenvironment [45,51,52]. microRNAs have also been shown as useful indicators of which patient groups may respond better to a particular treatment regimen. An example of this was shown for liver cancer patients, whereby miR-26 expression could be used to stratify patients for IFN treatment [53]. The full potential of microRNAs as prognostic factors awaits the results of larger prospective studies.

### The Therapeutic Application of MicroRNAs

As noted above, several microRNAs have been shown to be altered in disease states when compared to normal specimens. Whether this differential expression occurs as a consequence of the pathological state or whether the disease is a direct cause of this differential expression is currently unknown. Nonetheless, since microRNAs are deregulated in cancer, it is thought that normalization of their expression could be a potential method of intervention. In this vein, several therapeutic mechanisms have been put forth and are described below (Table 2).

**Table 2:** Strategies to Employ MicroRNAs in the Clinic

Strategy	Modulator	Delivery	Clinical Utility	References
Inhibition of mature microRNA cluster	microRNA sponge	Sponge plasmid vector	Silence oncomiR cluster	[60]

Inhibition of mature microRNA	2'OME-AMOs	RNA-Liposome Complex	Silence OncomiR	[94]

Inhibition of mature microRNA	2'MOE AMOs	Oligonucleotide-Liposome Complex	Silence OncomiR	[95,96]

Inhibition of pri-microRNA	AMOs	Oligonucleotide-Liposome Complex	Silence miR cluster	[97,98]

Inhibition of mature microRNA	LNA-antagomiR	Unconjugated	Silence OncomiR	[99]

Silence selected target	Synthetic microRNAs	Conjugation	Tumor Suppressor Function	[100,101]

#### Strategies for microRNA reduction

The rules of Watson and Crick base-pairing guide the binding of microRNAs to their target sites. In order to circumvent this interaction, anti-microRNA oligonucleotides (AMOs) have been generated to directly compete with endogenous microRNAs [54]. However, the ability of AMOs to specifically inactivate endogenous targets has been shown to be quite inefficient. Thus, several modifications of AMOs have been generated to improve their effectiveness and stability such as the addition of 2'-O-methyl and 2'-O-methoxyethyl groups to the 5' end of the molecule [55]. Studies have shown that targeting of miR-21, a microRNA that is overexpressed in many cancer types, by such methods effectively reduced tumor size in a xenograft mouse model based on MCF-7 cells [56]. AMOs conjugated to cholesterol (antagomirs) have been also been generated and have been described to efficiently inhibit microRNA activity in-vivo [57]. In addition, locked-nucleic-acid antisense oligonucleotides (LNAs) have been designed to increase stability and have been shown to be highly aqueous and exhibit low toxicity in-vivo [58]. In gliomas, this method has been effectively used to completely eradicate miR-21 [59]. Another method for reducing the interaction between microRNAs and their targets is the use of microRNA sponges. These sponges are synthetic mRNAs that contain multiple binding sites for an endogenous microRNA. Sponges designed with multimeric seed sequences have been shown to effectively repress microRNA families sharing the same seed sequence [60]. Although microRNA sponges perform as well as chemically modified AMOs in-vitro, their efficacy in-vivo remains to be determined.

Although these oligonucleotide-based methods have been shown to work, they do elicit off-target side effects and unwanted toxicity. This is due to the capability of microRNAs to regulate hundreds of genes. A strategy called miR-masking is an alternative strategy designed to combat this effect. This method utilizes a sequence with perfect complementarity to the target gene such that duplexing will occur with higher affinity than that between the target gene and its endogenous microRNA. The caveat of this approach is that the choice of target gene must be specific in order to effectively reduce the interaction. This gene-specific, microRNA interfering strategy has been shown to reduce the activities of miR-1, miR-133 and miR-430 in several model systems [61,62]. Another strategy to increase specificity of effects is the use of small molecule inhibitors against specific microRNAs. Azobenzene, for example, has been identified as a specific and efficient inhibitor of miR-21 [63]. Although the effectiveness of such inhibitors awaits exploration in-vivo, they are potentially promising tools for cancer therapy.

#### Strategies to overexpress microRNAs

Elevating the expression of microRNAs with tumor suppressive roles is a strategy to restore tumor inhibitory functions in the cell. This can be achieved through the use of viral or liposomal delivery mechanisms [64,65]. Several microRNAs have been introduced to cells via this methodology, including miR-34, miR-15, miR-16 and let-7 [66-69]. Systemic administration of miR-26, a tumor suppressive microRNA in HCC, using adenovirus-associated virus (AAV) in an animal model of HCC, results in inhibition of cell proliferation and tumor-specific apoptosis [70]. This approach reduces toxicity since AAV vectors do not integrate into the host genome and eventually are eliminated. Although viral vector-directed methods show high gene transfer efficiency, they lack tumor targeting and residual viral elements can elicit immunonogenic effects. This has led to the development of non-viral methods of gene transfer such as cationic liposome mediated systems. These lipoplexes are promising, but they lack tumor specificity and have relatively low transfection efficiency when compared to viral vectors.

MicroRNA mimics have also been used to increase microRNA expression. These small, chemically modified double-stranded RNA molecules mimic endogenous mature microRNA. These mimics are now commercially available and promising results have been reported with systemic delivery methods using lipid and polymer-based nanoparticles [71-73]. Since these mimics do not have vector-based toxicity, they are promising tools for therapeutic treatment of tumors.

## Conclusions

As described above, there have been many new technological advances to utilize microRNAs as therapeutic tools. In order to fully achieve this however, conceptual and technical issues still need to be overcome. Since microRNAs can potentially inhibit many genes, a major hurdle to overcome is specificity. Partial complimentarity can lead to off-target gene silencing or up-regulation and thus undesired biological effects. Given the multi-gene targets of a single microRNA, the magnitude of an off-target association may be quite large. Thus, it remains important to comprehensively evaluate each specific microRNA-mediated therapy. Conversely, it may be useful to target multiple members of a gene family with a single microRNA. Such strategies are currently underway to design small multiple target artificial (SMART) microRNAs to simultaneously target members of a single gene family, such as E2F [74]. A more thorough understanding of microRNA biology and function will allow for more suitable strategies. Another issue that warrants future study is the efficiency of delivery of microRNA to specific sites. One needs to achieve a certain amplitude of target gene modulation and to maximize the number of cells that receive therapeutic microRNA at target sites. This effect also needs to be long-lasting with minimal toxicity to the recipient. Further advances in the area of drug delivery will no doubt improve upon the current tools of the trade.

Once a provocative finding in a worm-based model, microRNAs have now become a grand player in the field of biological science and clinical therapy. Research within the last few decades has significantly added to our knowledge of the biogenesis and function of microRNAs. These studies have shown that microRNAs play a large and key role in many aspects of cancer biology and that alteration of their expression can have profound effects on cancer phenotypes. The translation of these findings to in-vivo models and clinical studies will unquestionably lead to greater insight into their utility in clinical settings. The notion of microRNAs as therapeutic agents is in the first phases and is at the cusp of providing major advances in research and to enhancing the tools available to alleviate cancer.

## Competing interests

The authors declare that they have no competing interests.

## Authors' contributions

A.B. wrote the review; J.J. and X.W.W. provided constructive review of manuscript. All authors have read and approved the final manuscript.
